# Angiopoietin‐2 provides no incremental predictive value for the presence of obstructive coronary artery disease over N‐terminal pro‐brain natriuretic peptide

**DOI:** 10.1002/jcla.22972

**Published:** 2019-07-01

**Authors:** Wen Jian, Chang‐Hua Mo, Guo‐Liang Yang, Lang Li, Chun Gui

**Affiliations:** ^1^ Department of Cardiology The First Affiliated Hospital of Guangxi Medical University Nanning China; ^2^ Guangxi Key Laboratory Base of Precision Medicine in Cardio‐Cerebrovascular Diseases Control and Prevention Nanning China; ^3^ Guangxi Clinical Research Center for Cardio‐Cerebrovascular Diseases Nanning China

**Keywords:** angiopoietin‐2, biomarkers, coronary artery disease, diagnostic cardiac catheterization, NT‐proBNP

## Abstract

**Background:**

Using circulating biomarkers as a noninvasive method to assist the evaluation of coronary artery disease (CAD) is beneficial for reducing the unnecessary diagnostic cardiac catheterization. This study aimed to assess the predictive role of angiopoietin‐2 (Ang‐2) for the presence of obstructive coronary stenosis as compared with N‐terminal pro‐brain natriuretic peptide (NT‐proBNP) in patients with symptoms of CAD.

**Methods:**

The study enrolled 222 consecutive symptomatic patients who underwent elective diagnostic cardiac catheterization from July to December 2018. Blood samples were collected in the first morning after admission. The severity of coronary stenosis was assessed by coronary angiography. The obstructive CAD was defined as stenosis ≥50% of the left main coronary artery or stenosis ≥70% of a major epicardial vessel (left anterior descending artery, left circumflex artery and right coronary artery).

**Results:**

Patients with obstructive CAD (n = 120) had significantly higher levels of Ang‐2 and NT‐proBNP compared with those without. In multivariable regression analysis, only NT‐proBNP levels were independently associated with Ang‐2 levels. NT‐proBNP was superior to Ang‐2 as a predictor for the presence of obstructive CAD (NT‐proBNP, area under curve [AUC] = 0.733, vs Ang‐2, AUC = 0.626, *P* = 0.004). In multiple logistic regression analysis, NT‐proBNP, but not Ang‐2, was the independent predictor of obstructive CAD. The combination of Ang‐2 with NT‐proBNP did not provide the incremental value over NT‐proBNP alone.

**Conclusion:**

Serum Ang‐2 levels are associated with NT‐proBNP levels in patients suspected for CAD. NT‐proBNP is superior to Ang‐2 as a predictor for the presence of obstructive CAD. However, Ang‐2 does not further increase diagnostic accuracy on top of NT‐proBNP.

## 1 INTRODUCTION

Coronary artery disease (CAD) is the leading cause of death worldwide. For symptomatic patients in nonacute condition, elective cardiac catheterization and coronary angiography are widely applied in order to evaluate degree of coronary artery stenosis and determine the need for interventional therapy. Despite the high prevalence of CAD, the low diagnostic yield of cardiac catheterization in routine clinical practice is of great concern.^1^ Efforts for better risk stratification can not only lower the cost burden, but also avoid the procedural complications, the radiation exposure, and the renal injury by contrast agent. In the context of a patient with traditional risk factors, multiple noninvasive approaches have been used to assist the evaluation of obstructive CAD, such as stress testing, coronary computed tomographic angiography, and so on.^2^ However, the incremental value of these approaches is limited by the variable sensitivity and specificity. Furthermore, it would not be practical to apply these techniques to all the ones with suspected obstructive CAD in the general population. Thus, in order to avoid the unnecessary diagnostic cardiac catheterization, circulating biomarkers are of great potential value as a noninvasive method to assist the evaluation of CAD in addition to the known clinical risk factors.^3^, ^4^

Sustained ischemia due to coronary stenosis or occlusion induces the dysfunction of myocardial cells, resulting in the cardiac remodeling and subsequently heart failure. Angiogenesis is a self‐compensation process increasing myocardial microvascular network to relieve the hypoxic situation and maintain cardiac contractile function.^5^ Several growth factors are closely associated with angiogenesis such as angiopoietin‑1 (Ang‑1), angiopoietin‑2 (Ang‑2), and vascular endothelial growth factor (VEGF). Angiopoietin‐2 (Ang‐2), a context‐dependent antagonist through inhibition of Ang‐1–induced Tie2 phosphorylation, can lead to vascular destabilization and play an important role in angiogenesis.^6^, ^7^ Ang‐2, which is released from Weibel‐Palade bodies (WPB) of endothelial cells upon stimulations,^8^ can enhance new vessels branching and sprouting. Ang‐2 promotes angiogenesis in the context of VEGF, but inhibits that in the absence of VEGF.^9^

N‐terminal pro‐brain natriuretic peptide (NT‐proBNP), a marker secreted in response to elevated volume and pressure load as well as hypoxia, has been found to be elevated in stable CAD.^10^, ^11^, ^12^ Previous study has demonstrated that NT‐proBNP levels are associated with the severity of coronary stenosis, and combination of NT‐proBNP with exercise testing can improve the predictive accuracy for severe CAD in patients with stable angina.^11^ Even in patients with normal left ventricular function suspected for CAD, NT‐proBNP is also an independent biomarker predicting significant coronary stenosis (≥50%).^12^ Of note, growing evidence has shown the close relation between Ang‐2 and NT‐proBNP in multiple cardiovascular diseases. Ang‐2 levels progressively increase as the parameters of cardiac function decline in stable chronic heart failure (CHF), and the performance of Ang‐2 in predicting heart failure is similar to that of NT‐proBNP.^13^ In patients with congenital heart disease, the value of Ang‐2 as a biomarker detecting CHF is comparable to NT‐proBNP.^14^ However, Ang‐2 cannot predict 1‐year outcome independently of NT‐proBNP in CHF.^15^ Thus, this study aimed to assess the predictive role of Ang‐2 for the presence of obstructive coronary stenosis as compared with NT‐proBNP in patients with symptoms of CAD.

## 2 MATERIALS AND METHODS

### 2.1 Study population

The study enrolled 222 consecutive symptomatic patients who underwent elective diagnostic cardiac catheterization from July to December 2018. The patients with acute decompensated heart failure, pulmonary embolism, and aortic dissection were excluded, as were the patients with indications for emergency or urgent cardiac catheterization (unstable angina in high‐risk, acute myocardial infarction). In patients with elevated baseline hsTnI levels (>99th percentile upper reference limit), a repeat measurement later was conducted to confirm its stable status. The study protocol was reviewed and approved by the Human Research Ethics Committee of the First Affiliated Hospital of Guangxi Medical University, China. Written informed consents were obtained from all patients. The demographics and baseline clinical characteristics were recorded in the first day after inclusion. Results of coronary angiography (based on visual assessment) were recorded by experienced angiographers. The obstructive CAD was defined as stenosis ≥50% of the left main coronary artery, or stenosis ≥70% of a major epicardial vessel or their branches (left anterior descending artery, left circumflex artery, and right coronary artery). The number of stenotic coronary arteries (≥70%) was documented, and the left main trunk disease (≥50%) was defined as 2‐vessel disease.

### 2.2 Laboratory measurements

The baseline laboratory data were acquired from the hospital's database. Blood samples for Ang‐2 without anticoagulant were collected in the first morning after admission and were centrifuged at 2600 *g* for 10 minutes. The serum supernatant was removed and stored at −80°C until it was used for analysis. Ang‐2 concentrations were measured with commercially available enzyme‐linked immunosorbent assay kits (RayBiotech, Inc). NT‐proBNP was measured with the Elecsys proBNP II reagent kit (Roche Diagnostics GmbH). High‐sensitive troponin‐I (hsTnI) was measured with the ARCHITECT STAT high‐sensitive troponin‐I assay (99th‐percentile‐cutoff: 26 ng/L). Estimated glomerular filtration rate (eGFR) was calculated using CKD‐EPI**_(Scr‐CysC)_** Equation 16.

### 2.3 Statistical analysis

Continuous variables are presented as median (IQR, interquartile range) and were compared using the Student t test or the Mann‐Whitney U test, as appropriate. Categorical variables are described with counts and percentages and were compared using the chi‐square or the Fisher exact test. The normality of the numeric variables was assessed with the Kolmogorov‐Smirnov test. Given the skewed distribution, a base‐2 logarithmic transformation (Ang‐2, NT‐proBNP, hsTnI, etc) was applied. Correlation statistics were performed by using the Pearson correlation test, and multiple regressions were used to identify independent factors associated with the serum Ang‐2 levels. The traditional risk factors (age, BMI, hypertension, diabetes, and smoking) and the variables with a *P* value < 0.1 in the univariate analysis (gender, previous myocardial infarction [MI], previous percutaneous coronary intervention [PCI], eGFR, hsTnI, NT‐proBNP, and Ang‐2) were selected for the multivariate logistic regression analysis to evaluate the independent predictors of obstructive CAD. Receiver operating characteristic (ROC) curve analysis was used to determine the optimal cutoff value of Ang‐2 and NT‐proBNP to predict obstructive CAD. Comparisons between AUC were made using the method described by DeLong.^17^ Two‐sided *P* values of <0.05 were considered statistically significant. Analysis was done using SPSS, version 19, and MedCalc, version 18.2.1.

## 3 RESULTS

### 3.1 Characteristics of the study subjects

The baseline characteristics are summarized in Table 1. Among 222 subjects enrolled in this study, 120 subjects were found with obstructive CAD after the cardiac catheterization (42.5% had 1‐vessel disease, 23.3% had 2‐vessel disease, and 34.2% had 3‐vessel disease). There were more males in the CAD group compared with non‐CAD group (74.2% vs 56.9%, *P* = 0.007). The prevalence of smoking, diabetes, previous MI, and previous PCI in CAD subjects was higher than non‐CAD subjects. In addition, CAD subjects had higher levels of renal‐function parameters (serum creatinine [Scr], cystatin C [CysC], eGFR), left ventricular ejection fraction [LVEF], NT‐proBNP, hsTnI, and Ang‐2. No significant difference was found between groups in terms of age, BMI, hypertension, serum lipids, and creatine kinase MB form.

**Table 1:** Baseline characteristics

	Subjects without coronary stenosis ≥ 70% (n = 102)	Subjects with coronary stenosis ≥ 70% (n = 120)	*P*‐value
Age	60 (51‐66)	61 (53‐67)	0.56
BMI	24.82 (22.40‐26.35)	24.22 (21.88‐26.26)	0.68
Male gender	58 (56.9%)	89 (74.2%)	0.007
Hypertension	66 (64.7%)	72 (60.0%)	0.47
Diabetes	17 (16.7%)	38 (31.7%)	0.01
Smoking	30 (29.4%)	61 (50.8%)	0.001
Previous MI	8 (7.8%)	27 (22.5%)	0.003
Previous PCI	10 (9.8%)	31 (25.8%)	0.002
Systolic BP (mm Hg)	132 (122‐146)	130 (120‐143)	0.49
Diastolic BP (mm Hg)	76 (71‐83)	75 (67‐80)	0.17
Total cholesterol (mmol/L)	4.44 (3.75‐5.05)	4.54 (3.65‐5.24)	0.28
Triglycerides (mmol/L)	1.27 (1.00‐1.97)	1.33 (1.06‐1.87)	0.59
LDL cholesterol (mmol/L)	2.61 (2.06‐3.24)	2.66 (2.10‐3.41)	0.16
HDL cholesterol (mmol/L)	1.02 (0.89‐1.13)	1.00 (0.81‐1.19)	0.90
Creatinine (µmol/L)	75 (64‐88)	81 (71‐99)	0.003
Cystatin C (mg/L)	0.90 (0.80‐1.05)	1.02 (0.86‐1.22)	0.001
eGFR (mL/min/1.73 m^2^)	86 (76‐101)	78 (66‐96)	0.007
LVEF, %	69 (65‐73)	67 (62‐70)	0.002
NT‐proBNP (ng/L)	53 (20‐88)	140 (51‐438)	<0.001
hsTnI (ng/L)	4 (3‐9)	5 (10‐22)	<0.001
CK‐MB (U/L)	13 (8‐16)	13 (9‐17)	0.18
Ang‐2 (ng/L)	1757 (1417‐2303)	2118 (1705‐2963)	0.001
Number of stenotic arteries (≥70%)			
1	0 (0%)	51 (42.5%)	<0.001
2	0 (0%)	28 (23.3%)	<0.001
3	0 (0%)	41 (34.2%)	<0.001

Note

Data are displayed as median (IQR, interquartile range) or n (%).

Abbreviations: Ang‐2, angiopoietin‐2; BMI, body mass index; BP, blood pressure; CK‐MB, creatine kinase MB form; eGFR, estimated glomerular filtration rate; HDL, high‐density lipoprotein; hsTnI, high‐sensitive troponin‐I; LDL, low‐density lipoprotein; LVEF, left ventricular ejection fraction; MI, myocardial infarction; NT‐proBNP, N‐terminal pro‐brain natriuretic peptide; PCI, percutaneous coronary intervention.

### 3.2 The relations between serum Ang‐2 levels and clinical or biochemical parameters

**Figure 1:**
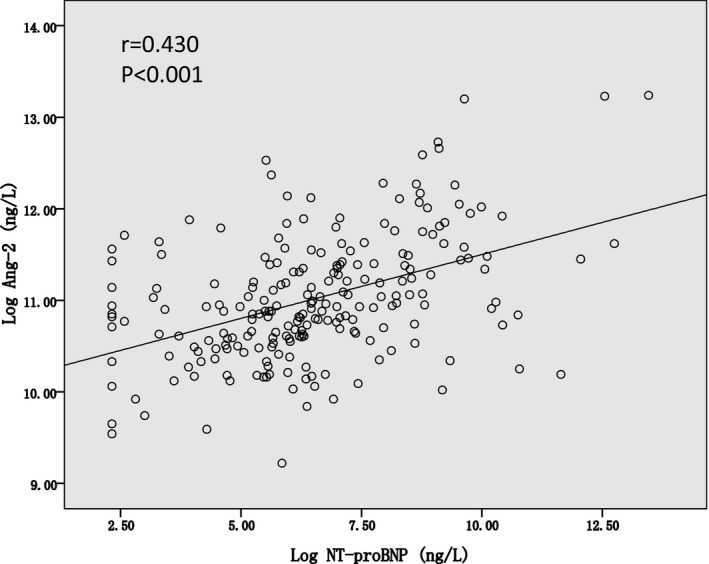
The association between NT‐proBNP and Angiopoietin‑2 levels (base‐2 logarithmic transformation) by Pearson correlation test

Ang‐2 levels were positively correlated with age (Pearson correlation coefficient [*r*_p_] = 0.155, *P* = 0.021), the number of stenotic vessels (*r*_p_ = 0.224, *P* = 0.001), hsTnI (*r*_p_ = 0.140, *P* = 0.045), and NT‐proBNP (*r*_p_ = 0.430, *P* < 0.001), but inversely correlated with eGFR (*r*_p_ = −0.202, *P* = 0.003) (Table 2). However, in a multivariable regression model (including age, BMI, smoking, hypertension, diabetes, the number of stenotic vessels, eGFR, hsTnI, and NT‐proBNP), only NT‐proBNP levels were independently associated with Ang‐2 levels (standardized *β* = 0.474, *P* < 0.001, Figure 1).

**Table 2:** Correlation of serum Ang‐2 (Log) with clinical and biochemical variables

Variables	Pearson correlation coefficient	*P*
Age	0.155	0.021
the number of stenotic vessels	0.224	0.001
hsTnI (Log)	0.140	0.045
NT‐proBNP (Log)	0.430	<0.001
eGFR	−0.202	0.003

Abbreviations: Ang‐2, angiopoietin‐2; eGFR, estimated glomerular filtration rate; hsTnI, high‐sensitive troponin‐I; Log, Log‐transformed; NT‐proBNP, N‐terminal pro‐brain natriuretic peptide.

### 3.3 Predictive value of Ang‐2 and NT‐proBNP for obstructive CAD

**Figure 2:**
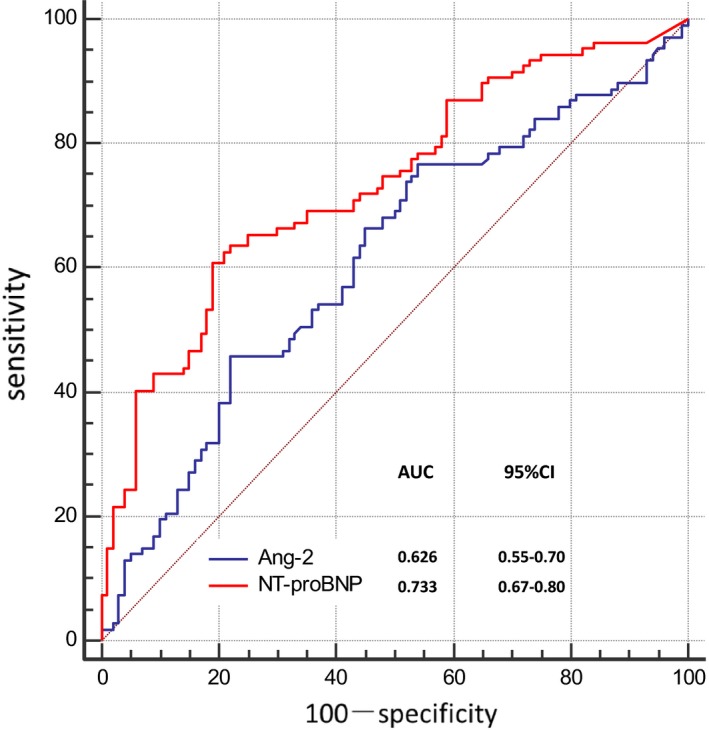
ROC curve analysis for predictive value of Ang‐2 and NT‐proBNP in detecting obstructive CAD

As shown in Figure 2, the ROC curve analysis revealed that using an optimal cutoff level of 2347 ng/L, Ang‐2 predicted obstructive CAD with 47% sensitivity and 78% specificity (area under curve [AUC], 0.626; 95% confidence interval [CI], 0.55‐0.70; *P* = 0.001). As for NT‐proBNP, the ROC curve showed a 61% sensitivity and 81% specificity with a cutoff level of 110 ng/L (AUC, 0.733; 95% CI, 0.67‐0.80; *P* < 0.001). NT‐proBNP could predict obstructive CAD better than Ang‐2 (AUC 0.733 vs AUC 0.626, *P* = 0.004). Furthermore, after adjusting with the covariates, multiple logistic regression analysis showed that NT‐proBNP, but not Ang‐2, was an independent predictor of obstructive CAD (Table 3). The combination of Ang‐2 with NT‐proBNP did not provide the incremental value over NT‐proBNP alone (AUC, 0.732 vs AUC, 0.733; *P* = 0.99).

**Table 3:** Binary logistic regression model in univariate and multivariate analysis for prediction of obstructive coronary artery disease

Variables	Univariate OR (95% CI)	*P*	Multivariate OR (95% CI)	*P*
Ang‐2 (Log)	1.86 (1.22‐2.83)	0.004	–	NS
NT‐proBNP (Log)	1.56 (1.33‐1.83)	<0.001	1.57 (1.32‐1.88)	<0.001
Male gender	2.18 (1.24‐3.84)	0.007	2.78 (1.39‐5.57)	0.004
Previous PCI	3.20 (1.48‐6.92)	0.003	2.81 (1.20‐6.58)	0.017

Note

The multivariate model included age, gender, BMI, hypertension, diabetes, smoking, previous MI, previous PCI, eGFR, hsTnI, NT‐proBNP, and Ang‐2.

Abbreviations: Ang‐2, angiopoietin‐2; BMI, body mass index; CI, confidence interval; hsTnI, high‐sensitive troponin‐I; MI, myocardial infarction; NS, nonsignificant; NT‐proBNP, N‐terminal pro‐brain natriuretic peptide; OR, odds ratio; PCI, percutaneous coronary intervention.

## 4 DISCUSSION

In this study, we found a close relation of Ang‐2 with NT‐proBNP in patients suspected for obstructive CAD. NT‐proBNP surpasses Ang‐2 as a predictor for the presence of obstructive CAD. However, Ang‐2 does not further increase diagnostic accuracy on top of NT‐proBNP.

Previous studies have reported the close relations between Ang‐2 and CAD. Serum Ang‐2 levels gradually increase with the advance of CAD,^18^ and PCI can reduce its levels.^19^ In patients with acute coronary syndrome, Ang‐2 is one of the top upregulated genes in the site of ischemic myocardium.^20^ With regard to the traditional risk factors of CAD, such as hypertension and diabetes, circulating Ang‐2 levels are elevated regardless of the vascular disease.^21^, ^22^

To my knowledge, only a small study (n = 70) before was conducted to demonstrate the value of Ang‐2 in predicting CAD (AUC = 0.722) despite lacking of NT‐proBNP measurement and a definition of ≥50% stenotic coronary artery.^23^ Our study extended the study subjects to all the patients suspected for CAD in the context of nonacute conditions admitted to our hospital. However, we observed both a mild diagnostic accuracy (AUC = 0.626) and a weak correlation between serum Ang‐2 levels and the number of stenotic vessels (*r* = 0.224), suggesting a limited value of Ang‐2 as a predictive biomarker for symptomatic patients in stable condition. The possible explanations may include the following: First, besides the role in angiogenesis, Ang‐2 can also regulate inflammation. Ang‐2, which is expressed weakly in resting quiescent endothelium, can be quickly released after a transition following endothelial activation.^6^, ^7^ In addition to hypoxia, the endogenous chemicals induced by inflammatory response (tumor necrosis factor‐α, reactive oxygen species, histamine, thrombin, etc) can activate the secretion of Ang‐2 from WPB.^8^, ^24^, ^25^ Thus, the relative quiescent endothelial system in the stable patients may account for the expression of Ang‐2 lower than ACS patients. Second, coronary collateral vessel development may have some effect on serum Ang‐2 levels, which was not analyzed in this study. Third, elevated concentrations of Ang‐2 have been reported under a broad range of diseases such as heart failure,^13^, ^14^, ^15^ microvascular disease,^26^ chronic kidney disease,^27^ tumor,^28^ and inflammatory disease.^29^, ^30^ Therefore, all these confounding factors may influence the predictive accuracy.

Chong et al reported that plasma Ang‐2 concentrations were correlated with LVEF in CHF.^31^ Eleuteri et al found Ang‐2 progressively increases as the cardiac function declines and is mainly associated with peak oxygen consumption in CHF.^13^ Lukasz et al reported that Ang‐2 is associated with NYHA class and ventricular dysfunction comparable to NT‐proBNP in CHF with congenital heart disease, and NT‐proBNP (AUC = 0.784) is superior to Ang‐2 (AUC = 0.656) for identifying patients with severely limited cardiopulmonary exercise capacity.^14^ In this study, we found that only NT‐proBNP levels were independently associated with Ang‐2 levels, even adjusting the variables which have been found to be related to Ang‐2 levels such as eGFR,^27^ hypertension,^21^ and diabetes.^22^ This indicates that at least in this study population, cardiac function may be the most prominent factor influencing serum Ang‐2 levels. In turn, despite enhancing angiogenesis, high level of Ang‐2 may antagonize the protective effect of Ang‐1 leading to cardiac injury, fibrosis, and remodeling.^32^, ^33^

Most previous studies have focused on the prognostic value of NT‐proBNP in predicting adverse cardiovascular outcomes.^10^, ^15^ For noninvasive diagnosis of CAD, more efforts based on the large studies are required. In patients with acute coronary syndrome, NT‐proBNP is associated with the severity of coronary artery stenosis.^34^ In patients with stable angina, NT‐proBNP can predict the extent of CAD.^11^ Even in patients with normal left ventricular function suspected for CAD, NT‐proBNP is also an independent biomarker predicting significant coronary stenosis.^12^ Consistent with their findings, we observed positive predictive value of NT‐proBNP. Despite the increasing evidence which has shown that Ang‐2 is involved in the development and progression of atherosclerosis,^35^ Ang‐2 does not further increase diagnostic accuracy on top of NT‐proBNP in this study. More efforts should be made to explore the precise cutoff point of NT‐proBNP in predicting obstructive CAD for clinical utility.

This study was limited by the single center with small sample size. The influence of drugs used prior to admission was not in consideration. Further large studies need to be done to confirm these findings.

## 5 CONCLUSION

Serum Ang‐2 levels are associated with NT‐proBNP levels in patients suspected for CAD. NT‐proBNP is superior to Ang‐2 as a predictor for the presence of obstructive CAD. However, Ang‐2 does not further increase diagnostic accuracy on top of NT‐proBNP.
